# Key Informant Views of the Determinants of Child Labour Maltreatment

**DOI:** 10.3390/children11060708

**Published:** 2024-06-08

**Authors:** Md. Abdul Ahad, Yvonne Karen Parry, Eileen Willis, Shahid Ullah

**Affiliations:** 1 Department of Rural Sociology and Development, Sylhet Agricultural University, Sylhet 3100, Bangladesh; ahad0005@flinders.edu.au; 2 College of Nursing and Health Sciences, Flinders University, Adelaide, SA 5042, Australia; yvonne.parry@flinders.edu.au; 3 School of Nursing, Midwifery and Social Sciences, Central Queensland University, Bundaberg, QLD 4701, Australia; 4 College of Medicine and Public Health, Flinders University, Adelaide, SA 5042, Australia; shahid.ullah@flinders.edu.au

**Keywords:** child labourers, maltreatment, qualitative study, Bangladesh, determinants

## Abstract

(1) Background: The maltreatment of child labourers is a major public health concern. There is a dearth of research in Bangladesh on the intentional maltreatment of child labourers. This study explored the risk factors for the maltreatment of child labourers in rural Bangladesh based on the knowledge and understanding of experts; (2) Methods: Seventeen interviews were conducted with experts who were sampled using a purposeful approach. A thematic analysis was performed to analyse data using NVivo; (3) Results: Child labourers were exposed to maltreatment due to their demographic, their young age, dropping out of school, health complications, and excessive dependency on employers. Socio-cultural adversities such as corporal punishment practices, social stratification, and family disorganization pose risks of experiencing maltreatment. Economic poverty is also a factor. Child labourers were found to be victimized if they worked in violence prone sectors. Significantly, the unregulated market structure and the lack of monitoring has also led to the exploitation of children in the workplace. Gaps in public policies were also identified as risk factor for the maltreatment of child labourers; (4) Conclusion: There is a need for adequate evidence-based research on the determinants of the maltreatment of child labourers to formulate adequate policy.

## 1. Introduction

Child labour and maltreatment are serious social and public health concerns, particularly in low-income countries. Children who are labourers and who are maltreated tend to have significantly negative physical, emotional, cognitive, and social outcomes throughout their lives [1,2,3]. There are no universally agreed definitions of child labour; however, the definitions can be grouped into two categories: market-based work, which includes work for wages in formal or informal sectors, and work in the home, farm or family enterprise, or the production of goods and services that are generally not traded for monetary purposes. Both are detrimental to the potential and dignity of children as well as to their physical and psychological well-being if carried out for an extended number of hours [4]. According to the Bangladesh Labour Act 2006, children aged below 14 years involved in paid or unpaid labour, or who engage in hazardous work who are under 18 years of age, are considered to be child labourers [5].

Compared to other continents, child labour is highly prevalent in South Asian countries. After Nepal, Bangladesh has the highest percentage of child labourers, aged 5–17 years, (5 million) among South Asian countries based on its population density [6]. The National Child Labour Survey, 2013 reported that there are 1.7 million child labourers in Bangladesh [7]. The highest proportions are engaged in the agricultural sector followed by the service sectors. Research indicates that more than 0.42 million children are employed as domestic child labourers in Bangladesh [8]. Several prior studies have argued that these children are more likely to suffer abusive treatment or maltreatment than any other group [9,10]. Child maltreatment refers to any form of malice, harm, or offense that inflicts injuries, humiliation, embarrassment, or fear on a child through intentional or unintentional conduct [11]. In addition to the challenges posed by working long hours, a further experience of maltreatment compounds their social and health vulnerability. This study focused specifically on the determinants of maltreatment of child labourers.

Numerous studies have separately examined the factors contributing to child labour and child maltreatment, indicating that there are several push and pull factors involved, rather than only a single factor [12,13,14,15]. The first and foremost fact about child labour is poverty, which pushes unprivileged children into this form of exploitation. The pervasiveness of child labour and maltreatment is also characterised by a higher proportion of illiteracy and low-income families and is found in regions with abnormally high rates of unemployment [12,13,14,15]. Additionally, research has shown that child labourers’ internal vulnerability factors, including their young age, school dropout status, extended working hours, and low wages, significantly impact their levels of abuse [10,15,16,17]. Further, disorganized family environments such as those with domestic violence, parental separation, drug abuse, etc., often contribute to the maltreatment of these children. In many South Asian countries, culturally accepted practices of sending children to work in order to instil responsibility in them, as well as harsh discipline, also serve as potential risk factors for the maltreatment of child labourers [14,16,18]. Intergenerational childhood maltreatment is another factor. Children who witness or experience maltreatment may internalize violent behaviour as a normative means of resolving conflicts or exerting control, perpetuating a cycle of violence within families and communities when they become parents or employers themselves [11,19]. Additionally, the limitation and the absence of laws are thought to increase the risk of children being maltreated in the workplace. For example, the Labour Act 2006 is not applicable to the informal sector in Bangladesh [20]. Further, unlike most South Asian countries that ratified the ILO Convention on Minimum Age in previous years, to combat child labour and abuse, Bangladesh only ratified it in 2022 [21]. Moreover, there is no international agreement that ensures the enforcement of laws protecting child labourers from maltreatment [22]. Research into the maltreatment of child labourers is primarily conducted in urban settings [23,24]. Consequently, there is a lack of valid data regarding child maltreatment in the rural context, making it difficult to design, implement, and enforce policies to protect these children. This study explored the risk factors or determinants of maltreatment of rural child labourers, drawing on the views of experts.

## 2. Materials and Methods

In this study, we employed the key informant methodology [25]. In this approach, individuals (key informants) who are known to have expertise on the topic under investigation are sought out and interviewed [26]. There is an assumption that each expert will possess evidence-based knowledge of the maltreatment of child labourers (aged 5–18 years) beyond the lived experience of ordinary people, which is often the focus of qualitative research [27]. Homburg and colleagues note that there are three key factors to be considered when selecting key informants for interviews: the hierarchical structure of the organisation and where the informant is positioned within this structure, the specific role the individual has in the organisation, and the duration of their tenure [28]. Considering these criteria, we were able to identify suitable experts for this study.

### 2.1. Participants

Using purposeful sampling we contacted seventeen experts with research and administrative experience in child protection; this number was chosen in accordance with Morse’s recommendation that saturation can be achieved by 10 to 12 interviews [29]. A two-stage recruitment process was used. At the threshold level, recruitment commenced by visiting the websites of relevant organizations and reviewing their expertise in child protection. A second phase consisted of contacting the experts via their respective line managers or, in the case of academics, directly. Upon consent, the experts were contacted directly via email for an interview and with the purpose of the research explained. The relevant experience of these key experts is outlined below; notably, their experience extends beyond the lived experience of on-the-ground research, clinical or journalistic investigations, or involvement in policy formulation. See Table 1.

### 2.2. Procedure

Based on the study objectives, a semi-structured interview schedule was developed and validated after two pilot interviews. Participants were interviewed via the Internet platform, TEAMS, in Bengali or English at a time that was convenient for them, as it was not feasible for the key investigator to travel to Bangladesh during the COVID-19 pandemic. In accordance with the guidelines of the National Health and Medical Research Committee (NHMRC), all interviews were audiotaped and stored in a secure and password-protected location, and informed consent was obtained from all 17 experts [30]. All interviews conducted in the Bengali language were translated into English verbatim and transcribed before being uploaded to NVivo software. A pseudonym was assigned to each participant in order to ensure confidentiality and anonymity. The interviews were conducted between March and July 2021.

### 2.3. Analysis

Transcripts of the interview sessions were transcribed verbatim by a recognized transcription service. In order to ensure consistency and accuracy, the research team reviewed the interview transcripts several times. A thematic analysis was conducted using an inductive and interpretive approach [31]. As recommended by Braun and Clarke, we followed the steps including that the transcripts should be read and reread; this was followed by coding the specific objective-based evidence, constructing preliminary themes based on the identified robust codes, reviewing and reorganizing the preliminary themes, and defining, naming, and reporting on the reviewed themes [31]. The generation of themes was discussed within the research team, two of whom have expertise in qualitative research and child maltreatment studies to review and validate the emerged themes. NVivo software version 12 was used to conduct the thematic analysis. 

## 3. Results

The data identified from the key informant interviews developed five overarching dimensions as the determinants of child labour maltreatment.

### 3.1. Theme 1: Children and Interactional Factors

The children’s ontogenetic traits were identified as the major determinants of child maltreatment. This overarching theme related to the child labourers’ young age, gender, adverse health and disability characteristics, failure to access education, and dependency on employers at the workplace, which make them vulnerable to maltreatment. Several expert participants reported that due to their younger age, the children often fail to perform duties and are helpless in protecting themselves. One participant stated that

“…because they are young, they are incapable of doing the task. That is why, I think, the issue of the risk of childhood victimisation has increased”.

(G3)

Participants further argued that in patriarchal social systems, girls are often discriminated against. Additionally, these experts were well aware of the way violence against male child labourers was ignored. They believe that violence against female child labourers received more media attention than that against boys. One participant quoted that

“…various news media and reports predominantly highlight the violent incidences against female child labourers neglecting that (perpetrated) against the male child workers”.

(J2)

Expert personnel further argued that child labourers who have a congenital disability or are physically incapacitated often fail to perform duties on time. This puts them at risk of abuse and exploitation. One academic highlighted that

“…across the Indian subcontinent, children, in particular, with physical or intellectual disabilities are not normally accepted. Most family members consider them a burden”.

(A2)

Moreover, child labourers often suffer from various mental health disorders including depression, stress, anxiety, aggressive behaviour, etc. One paediatrician stated that these conditions led to maltreatment:

“[Some] suffer from various forms of mental depression, some suffer from constant anxiety—…… again they are being maltreated for not being able to work given their emotional state”.

(P1)

Key participants further stressed that the dropout rate from school is higher among child labourers in Bangladesh, which they highlighted as a causative factor of child maltreatment. One respondent explained the following:

“Family members continued beating or maltreating for the less dynamic traits of child labour such as their ignorance or dropping out of school, incompetence, misconduct”.

(G3)

Participants further suggested there is a need for child labourers to receive training to protect themselves, as pointed out by a respondent:

“…employers in the informal sector are reluctant to provide training. Consequently, the children make mistakes and experience abusive behaviour because of being unskilled”.

(G1)

The majority of the interviewed experts said that a substantial number of child labourers in Bangladesh were dependent on employers for their daily necessities as they lived with them, rather than with their parents. Supervision of workers in many informal workplaces is manipulatively time oriented, where the risk of experiencing maltreatment is high. Living with one’s employer also puts the child at risk of physical and sexual abuse. One academic noted that

“…following a shift, they usually live in the employer’s facilities. It may be difficult to find enough employers in the informal economy who are morally educated or socially superior… In this situation, it is very likely that children will be physically or sexually abused”.

(A1)

### 3.2. Theme 2: Socio-Cultural Challenges

The second theme identified the socio-cultural challenges. Participants argued that due to the incorporation of traditional practices within mainstream society, culturally abusive behaviours have long been accepted in society. The majority of key informants stressed that that the practice of corporal punishment in rural areas is deemed culturally appropriate in Bangladesh. One participant highlighted the following:

“We have a cultural belief about child labourers that if we punish them, they will be more productive in the home and workplace settings”.

(G1)

A participant further highlighted the class differences as a factor that results in forms of social stratification based on poverty and geographic locality. People are intolerant of low-status segments of society with child labourers being viewed as inferior. One participant said that

“…The upper class suppresses the lower class in society. If I were an owner, I will suppress my domestic helpers—children involved in labour”.

(P1)

The chaotic nature rooted in some family environments is also a factor behind child maltreatment. Participants argued that if children are not safe at home, where else can they be secure? Participants posited that the increasing pattern of interpersonal conflicts inside families forces children into labour where they are at risk of maltreatment. This is reflected in a quote from one participant:

“If you look at the recent reports, you can see that child maltreatment has also increased, which is actually a manifestation of domestic violence”.

(G4)

The nature of intolerance is also presumed to have arisen given the countries historical and cultural activities. Participants assumed Bangladesh’s past history of violence is part of the culture. Participants also were critical of the country’s political instability and volatile situation in the socio-cultural domain, which resulted in an inability to bring offenders to justice. In these cases, many people do not consider child maltreatment an act of violence, hence, they continue to abuse children. One key informant noted the following:

“I think—it’s just an assumption of mine that when people experience or witness severe violence, or murder, they try to ignore the less severe issues such as child abuse, or sexual harassment”.

(A3)

### 3.3. Theme 3: Economic Poverty and Child Maltreatment

Participants across all the interviews pointed out the financial strain on households is a significant determinant of child labour and exposure to maltreatment. They stressed that financial strain prompts many unemployed parents to send their children to work, where they are exposed to a range of intentional abuse and neglect. Key informants used the term “delay discounting” indicating that parents are more concerned about immediate short-term financial gains rather than the long-term value of schooling, noting the following:

“If they could look at future productivity, they would not send them to work. it’s not economically sensible…”.

(A3)

They also argued that employers often take advantage of the parents’ financial strain by enticing their children into labour. This has led children to be victimized both at home and in the workplace. The scenario is reflected in the quote below:

“The employer says, if I had not given them a chance to work, they would have died without food. […..] To send children in labour, they exhibit impulsive behaviour toward children”.

(NGO3)

### 3.4. Theme 4: Violence-Prone Informal Sectors and Structure

A noteworthy number of respondents noted that violence towards child labourers was concentrated in the informal sectors. Workplaces in the domestic and agricultural sectors are unsafe venues for children. Participants especially highlighted the work structure in the domestic sector. They stressed that domestic child labourers are encased by the four walls, where nobody can hear their voices. The experience of continuous exposure to violence desensitizes them to its effects, leading to the normalization of violence as a part of their daily lives. This normalization can perpetuate a cycle of violence. One participant stated that:

“…my experience is first and foremost with domestic child labourers outside—they suffer physical ill-treatment inside the four walls of private homes because of the few mistakes they make”.

(G3)

Participants further highlighted that within the rural informal sectors, such as small-scale agricultural establishments, often legal instructions and orders are not adhered to, which means they are hidden from public surveillance. It is therefore more likely that perpetrators will engage in abusive practices in these areas. This was noted as follows:

“……there is less scope for law enforcement or action by the government—what I said is, 95% of child labour is in the informal sector, here, there is no scope for government law enforcement”.

(G1)

### 3.5. Theme 5: Gaps in Public Policy Planning

Several participants noted that the government of Bangladesh has failed to mandate specific actions at the policy level in regard to child protection. They stressed that the constitution of Bangladesh does not explicitly mention child maltreatment or provide comprehensive protections against it. However, there are flaws in existing legal processes for handling child protection cases in Bangladesh, which may create barriers in accessing justice and seeking redress for instances of maltreatment. One key informant said the following:

“If we look at our country context, we will find that the severity of punishment for these crimes is not what it should be. Our social and judicial system does not punish them properly”.

(J1)

Participants further criticised the government inadequacy in framing legal entities related to child protection in rural areas. They suggested that child development monitoring centres in rural marginalised areas could address the problem of violence against children but are thin on the ground. Where this is not carried out, it perpetuates the rural culture of impunity and encourages continued exploitation of child labourers. This case is reflected in a quote of an academic:

“There are many agencies in the urban areas and those dealing with the social violence that we are talking about, but they are completely absent in the rural area”.

(A2)

They also noted that poor implementation of child protection laws is a barrier. There are some child protection laws in the country, but the ineffective enforcement mechanisms undermine their usefulness. Hence, they think that in most cases the real culprits are often acquitted, with the plaintiff suffering through various administrative hurdles.

Participants also stated that community monitoring and support programs are not yet adequate to protect vulnerable children. A number of participants said that socially disadvantaged children are not protected under social safety net programs. In the absence of social safety nets such as healthcare, childcare, and cash transfer programs, families may be forced to rely on child labour as a coping mechanism during times of economic hardship or crisis, which may leave them exposed to exploitation and abuse. One administrator stated the following:

“In some cases, where there are no working people in their family and children are engaged in labour, it may be possible to bring them under the social safety net programs to protect them—since the monitoring mechanism of the government is not so strong…but they lack resources”.

(G1)

## 4. Discussion

In this study, several overarching dimensions have been identified with respect to the critical individual and socioeconomic, cultural, and institutional factors that contribute to child labour maltreatment. Younger children, particularly those below the legal working age, are more vulnerable to maltreatment in workplace settings due to their physical, emotional, and cognitive immaturity [32]. Several empirical studies show that older children have a reduced risk of being abused and neglected than younger children [33,34,35]. According to the Bangladesh Bureau of Statistics (BBS), a substantial proportion of Bangladeshi child labourers are between the ages of 6 and 11 years [7]. They are considered a profitable asset in the labour market due to their low wages. However, employers prefer to hire young children in order to have greater control over them [22,36]. Hadi [10] also found that younger children are at a higher risk of experiencing abuse and neglect in Bangladesh. Studies have revealed that younger girls generally have an increased risk of abuses and exploitation over boys [32,35]. Similar to the views of key informants, prior studies have found that since girls are less likely to protest abusive employer behaviour, they are preferred to boys in the informal labour market. Girls are mostly recruited in third-party domestic households, where they work for long hours and may experience verbal, physical, or sexual abuse inside of the four walls of the home [36,37].

A further concern expressed by participants in this study was the children’s lack of access to education or failure to continue at school, which they cite as notable factors affecting child labour and their experience of abuse at work. In Bangladesh, only primary schooling is free and compulsory for everyone. Children of poverty-afflicted parents are often sent off to labour instead of attending secondary schooling. There is evidence that child labourers in Bangladesh who are out of school are more likely to be maltreated than children who are enrolled in school [9]. As noted by Lakhdir and colleagues [11], children without formal education are at an increased risk of verbal abuse, which is in accordance with the perspectives of key informants in this study. The pattern of labour and long working hours often results in child labourers suffering from various health hazards including malnutrition, physical injuries, various infectious diseases, and emotional disorders, etc. [32]. A key informant in this study also stressed that child labourers with these physical or psycho-somatic illnesses are at high risk of maltreatment within the workplace. As per the view of the key informants, those child labourers who are dependent on employers for their living arrangements are also at risk. Prior studies have shown that child labourers who sleep overnight at the workplace are more likely to experience physical maltreatment [33]. Banday and coauthors noted that child labourers living in employer-provided accommodation are routinely expose to physical and psychological abuse [17].The vulnerabilities of child labourers are further exacerbated due to the various socio-cultural forces and beliefs. There is a cultural belief and practice in some developing countries that children should engage in labour in order to develop their skills at an early age [22]. Key informants in this study identified this as a potential determinant of child labour. They further supposed that corporal punishment as a disciplinary practice is one of the most important cultural determinants of child labour maltreatment. Similar to the key informants’ views, numerous studies have also demonstrated that child maltreatment, particularly corporal punishment, is widely accepted and practiced in many countries, especially in the South Asia region [14,18,38]. This is despite the Bangladesh Government prohibiting all forms of corporal punishment in all educational institutions [39]. It is time to prohibit the corporal punishment of children practiced as a disciplinary act in all settings.

Bangladesh has a higher rate of domestic violence than many other Asian countries [40]. Several informants in this study pointed out that domestic violence has a direct correlation with child abuse. Typically, child labourers are members of low socio-economic households and are likely to find themselves in conflict with their family members due to unemployment and a low income [16,41]. Participants pointed out that the country has experienced wars and political instability at different points in its short history, which may have contributed to the intolerant attitude of its citizens towards children. Gibson [42] also argued that the more confrontation with wars and conflicts, or unstable political situations, the greater the likelihood of living in less-tolerant communities.

Child labour is rooted in economic insecurity within households. Similar to the current study, several prior studies have also noted that parental unemployment, debt, financial strain, or income–expense disparity in households leads parents to send their children to work as labourers to supplement the family income [13,22,23]. In poverty-stricken countries, such as Bangladesh, where there is little public financial support for vulnerable children and families, it is common for unemployed and impoverished parents to use their children for short-term benefits [14]. Employers also exploit poor families by encouraging parents to send their children to work [43,44]. Therefore, maltreatment towards these children is primarily influenced by economic crises. This study identified that a substantial proportion of children are working in the informal sector, which is not covered by the National Labor Acts, 2006) in Bangladesh as well as not being regulated by the core departments of government, making it easier for employers to exploit child labourers without facing legal repercussions [20,45]. Nearly all child labourers in Bangladesh are employed in the informal labour markets, where the nature of employment entails the possibility of abuse and exploitation [37]. Many are employed in the agricultural sector, and hence outside the purview of government.

## 5. Limitations of this Study

This study interviewed expert professionals from many relevant government and non-government departments. In a small number of cases, their knowledge of the maltreatment of child labourers came from secondary sources. This raises issues on the trustworthiness of their knowledge, and importantly, suggests that they may be uninformed when it comes to policy design.

### Implications for the Practice

This study suggests that a number of social and public health measures should be actioned to eliminate child labour maltreatment. As a preventive measure, social campaigns could be conducted to educate the public about the impact of maltreatment and neglect on children’s health and development. The awareness program could provide information regarding the impact of children dropping out of school, poor parenting skills, and domestic violence, and the risk of intergenerational cycles of child abuse and neglect. Bangladesh’s labour laws could be amended to include policies regarding the exploitation of children in the informal economy.

## 6. Conclusions

Maltreatment of child labourers is considered a global public health concern. The maltreatment of child labourers in Bangladesh is extremely common, particularly for children employed in the agricultural and domestic sectors. There are several studies on child labour in the literature, but the upward trend in violence affecting child labourers has only just begun to be explored by researchers. Steps need to be taken to bring the maltreatment and vulnerability aspects of these children under the child protection system by conducting research and reporting on these issues.

## Figures and Tables

**Table 1:** Study Participants.

Profession	Organization	Number of Participants
Academics and Researchers (Code A = academic or researcher)	Dhaka University (A1), Shahjalal University of Science and Technology (A2), Premier University (A3), North South University (A4), Khulna University (A5), and University of Northern Iowa (A6)	6
Government employees (G)	Ministry of Labor and Employment (G1), Ministry of Women and Children Affairs (G2), Ain O Salish Kendra (G3), Manusher Jonno Foundation (G4)	4
Non-government employees (NGO)	Global Vision (NGO1), UNICEF (NGO2), ILO (NGO3)	3
Journalists (J)	The Daily Kaler Khatna (J1), The Daily Star (J2)	2
Paediatricians (P)	Bangabandhu Sheikh Mujibur Rahman Medical University (P1), Osmani Medical College and Hospital (P2)	2

## Data Availability

The data cannot be made available due to restrictions of privacy.
